# Exploring the relationship between 24-2 visual field and widefield optical coherence tomography data across healthy, glaucoma suspect and glaucoma eyes

**DOI:** 10.1111/opo.13368

**Published:** 2024-07-26

**Authors:** Janelle Tong, Jack Phu, David Alonso-Caneiro, Jason Kugelman, Sieu Khuu, Ashish Agar, Minas Coroneo, Michael Kalloniatis

**Affiliations:** 1 Centre for Eye Health, University of New South Wales, Sydney, New South Wales Australia; 2 School of Optometry and Vision Science, University of New South Wales, Sydney, New South Wales Australia; 3 School of Medicine (Optometry), Deakin University, 3216 Waurn Ponds, Victoria Australia; 4 Faculty of Medicine, University of Sydney, Sydney, New South Wales Australia; 5 Concord Clinical School, Concord Repatriation General Hospital, Sydney, New South Wales Australia; 6 School of Science, Technology and Engineering, University of Sunshine Coast, Sunshine Coast, Queensland Australia; 7 Contact Lens and Visual Optics Laboratory, Centre for Vision and Eye Research, School of Optometry and Vision Science, Queensland University of Technology, Kelvin Grove, Queensland Australia; 8 Department of Ophthalmology, University of New South Wales at Prince of Wales Hospital, Sydney, New South Wales Australia

**Keywords:** glaucoma, optical coherence tomography, visual fields

## Abstract

**Purpose:**

To utilise ganglion cell-inner plexiform layer (GCIPL) measurements acquired using widefield optical coherence tomography (OCT) scans spanning 55° × 45° to explore the link between co-localised structural parameters and clinical visual field (VF) data.

**Methods:**

Widefield OCT scans acquired from 311 healthy, 268 glaucoma suspect and 269 glaucoma eyes were segmented to generate GCIPL thickness measurements. Estimated ganglion cell (GC) counts, calculated from GCIPL measurements, were plotted against 24-2 SITA Faster visual field (VF) thresholds, and regression models were computed with data categorised by diagnosis and VF status. Classification of locations as VF defective or non-defective using GCIPL parameters computed across eccentricity- and hemifield-dependent clusters was assessed by analysing areas under receiver operating characteristic curves (AUROCCs). Sensitivities and specificities were calculated per diagnostic category.

**Results:**

Segmented linear regression models between GC counts and VF thresholds demonstrated higher variability in VF defective locations relative to non-defective locations (mean absolute error 6.10–9.93 dB and 1.43–1.91 dB, respectively). AUROCCs from cluster-wide GCIPL parameters were similar across methods centrally (*p* = 0.06–0.84) but significantly greater peripherally, especially when considering classification of more central locations (*p* < 0.0001). Across diagnoses, cluster-wide GCIPL parameters demonstrated variable sensitivities and specificities (0.36–0.93 and 0.65–0.98, respectively), with the highest specificities observed across healthy eyes (0.73–0.98).

**Conclusions:**

Quantitative prediction of VF thresholds from widefield OCT is affected by high variability at VF defective locations. Prediction of VF status based on cluster-wide GCIPL parameters from widefield OCT could become useful to aid clinical decision-making in appropriately targeting VF assessments.

**Supplementary Information:**

The online version of this article (doi:10.1111/opo.13368) contains supplementary material, which is available to authorized users.

## Key points

- The use of a novel widefield optical coherence tomography protocol enabled the exploration of the links between co-localised retinal data and 24-2 visual field data across the glaucoma spectrum.
- Quantitative relationships between estimated ganglion cell counts and 24-2 thresholds were comparable to existing models at non-defective locations, but affected by high variability at defective locations, limiting predictive ability in disease.
- Comparisons between binarised structural and visual field results revealed reasonable specificities overall, suggesting potential use in targeting visual field assessments, although low sensitivities indicate persistence of some structure–function discordance.

## INTRODUCTION

Glaucoma remains a complicated disease to diagnose, requiring clinicians to collate and holistically interpret data from numerous aspects of the clinical examination. An additional complexity is the heterogeneity of glaucoma presentations; while classically glaucoma presents with concordant damage in the ocular structures and visual function, this so-called structure–function concordance is not always observed, which can contribute to diagnostic ambiguity between clinicians.^1,2^

Part of the difficulty in assessing structure–function concordance may lie in the different retinal areas over which measurements of structure and function are acquired. Clinically, visual function is most commonly assessed using standard automated perimetry with the 24-2 test pattern,^3,4^ capturing data over approximately 54° of the retina, while scan protocols on commercially available optical coherence tomography (OCT) instruments acquire peripapillary retinal nerve fibre layer (RNFL) and macular inner retinal layer thicknesses across a maximum retinal area of 30°. More recent OCT protocols have enabled acquisition of data over a 12 mm × 9 mm area (approximately 41.67° horizontally × 31.25° vertically) and can detect glaucoma equivalent to or better than conventional OCT protocols.^5–8^ However, this widefield protocol still does not capture data from retinal locations corresponding to up to 50% of 24-2 visual field (VF) test locations.^9^ Additionally, investigations of this OCT protocol have focused on the RNFL, whose trajectory can be influenced by various demographic and optic disc-related parameters and be prone to excessive inter-individual variability.^10–13^

Issues related to scan size could be addressed using OCT protocols capturing data over larger retinal areas. A recently described widefield OCT protocol measures 55° horizontally × 45° vertically and focuses on the ganglion cell-inner plexiform layer (GCIPL), thereby bypassing variability introduced by RNFL trajectory. Comparisons of co-localised GCIPL thickness between this and conventional OCT protocols have demonstrated equivalence.^14,15^ Using OCT data that can be reliably acquired over the retinal area sampled using the 24-2, a unique opportunity presents to explore directly the relationship between retinal structure and function over areas of interest in glaucoma.

Seminal models describing the relationship between retinal structure and visual function have traditionally incorporated ex vivo retinal ganglion cell (GC) or axon data as the structural parameter, which are unfeasible to measure from clinical data sets. The ability to estimate GC counts from OCT was first described by Raza and Hood,^16^ who derived volumetric GC cell densities from histological GC density data and macular ganglion cell layer (GCL) thickness.^17^ These data could subsequently be applied to calculate approximate in vivo GC counts, given a known macular GCL thickness and retinal area. Since it was first reported, this method has been extended to utilise the GCIPL,^18^ corresponding to the GCs and their dendritic processes.^19,20^ Estimation of cellular parameters from in vivo OCT measurements enables more direct comparisons of structure–function relationships from clinical cohorts to established models using histological GC data, such as that described by Garway-Heath et al.^21,22^

The present study sought to explore the link between structural parameters derived from GCIPL measurements acquired using widefield OCT and clinical 24-2 VF data across a spectrum of healthy, glaucoma suspect and glaucomatous eyes. First, quantitative structure–function relationships were derived by comparing pointwise GC counts estimated from widefield OCT-derived GCIPL thickness measurements to VF threshold data. These relationships were subsequently compared to the Garway-Heath et al.^21^ model to determine the validity of global structure–function relationships derived from the novel widefield OCT protocol. Second, to identify the level of concordance between widefield OCT and 24-2 VF data using approaches analogous to Hood et al. and assessments of structure–function concordance in clinical practice,^23^ the agreement between metrics derived from GCIPL thickness measurements and VF data binarised to within or outside of normative limits was assessed. It was hypothesised that quantitative structure–function models would be comparable to the Garway-Heath et al. model, and high concordance between binarised widefield OCT and VF results would be observed, thus demonstrating the potential clinical utility of widefield OCT in the accurate identification of structure–function concordance across the glaucoma spectrum.

## METHODS

### Participant recruitment

This prospective cross-sectional study recruited participants from patients attending the Centre for Eye Health, staff from the Centre for Eye Health and a private ophthalmology clinic, all located in Sydney, Australia. Participants provided written, informed consent prior to study participation. Ethics approval for this study was obtained from the University of New South Wales Human Research Ethics Advisory Panel, and the study adhered to the tenets of the Declaration of Helsinki throughout its duration.

Participants underwent at least one comprehensive examination as per Centre for Eye Health protocols,^24,25^ including slit-lamp biomicroscopy, funduscopic examination, central corneal thickness measurement (Pachmate; DGH Technology Inc., dghtechnology.com/), applanation tonometry, optic disc and macular OCT (Cirrus HD-OCT; Carl Zeiss Meditec Inc., zeiss.com/corporate/en/home.html and/or Spectralis OCT; Heidelberg Engineering, heidelbergengineering.com/int/) and visual field (VF) assessment using the 24-2 Swedish Interactive Threshold Algorithm (SITA) Faster paradigm (Humphrey Field Analyser; Carl Zeiss Meditec Inc., zeiss.com/corporate/en/home.html). The results from this examination were holistically assessed to diagnose participants as either:

- **Healthy**, when there was no optic nerve pathology, normal VF results and intraocular pressures (IOPs) < 22 mmHg in both eyes.
- **Glaucoma**, if there were characteristic signs of optic nerve head and peripapillary damage requiring existing or initiation of IOP-lowering treatment, including but not limited to focal or widespread thinning of the neuroretinal rim, an enlarged cup or RNFL defects visualised funduscopically or on OCT, irrespective of the pretreatment IOP or VF status.
- **Glaucoma suspect** when fitting neither of the categories described above, which may include any combination of elevated IOP or abnormal optic nerve head appearance, imaging or VF results. Participants in this category did not demonstrate sufficient evidence of glaucoma to warrant IOP-lowering treatment.

In healthy and glaucoma suspect participants, diagnoses were determined via consensus between the examining optometrist and another clinician, which varied between a senior optometrist, a general ophthalmologist and a glaucoma specialist ophthalmologist.^25,27^ Meanwhile, the diagnoses of glaucoma participants were all confirmed by a glaucoma specialist ophthalmologist.

Additional inclusion criteria across the glaucoma, glaucoma suspect and healthy participants were at least 20 years of age, spherical equivalent refractive error between +6.00 and −6.00 dioptres (D), no more than −3.00 D of astigmatism, no macular pathology in the included eye and a reliable 24-2 VF result in the included eye, based on a false-positive criterion of <15% and no obvious VF artefacts such as seeding point error.^28^ Where only one eye met these criteria, that eye was selected to undergo further analyses, while if both eyes met the inclusion criteria, then one eye was selected at random.

### Widefield OCT data acquisition and processing

**FIGURE 1:**
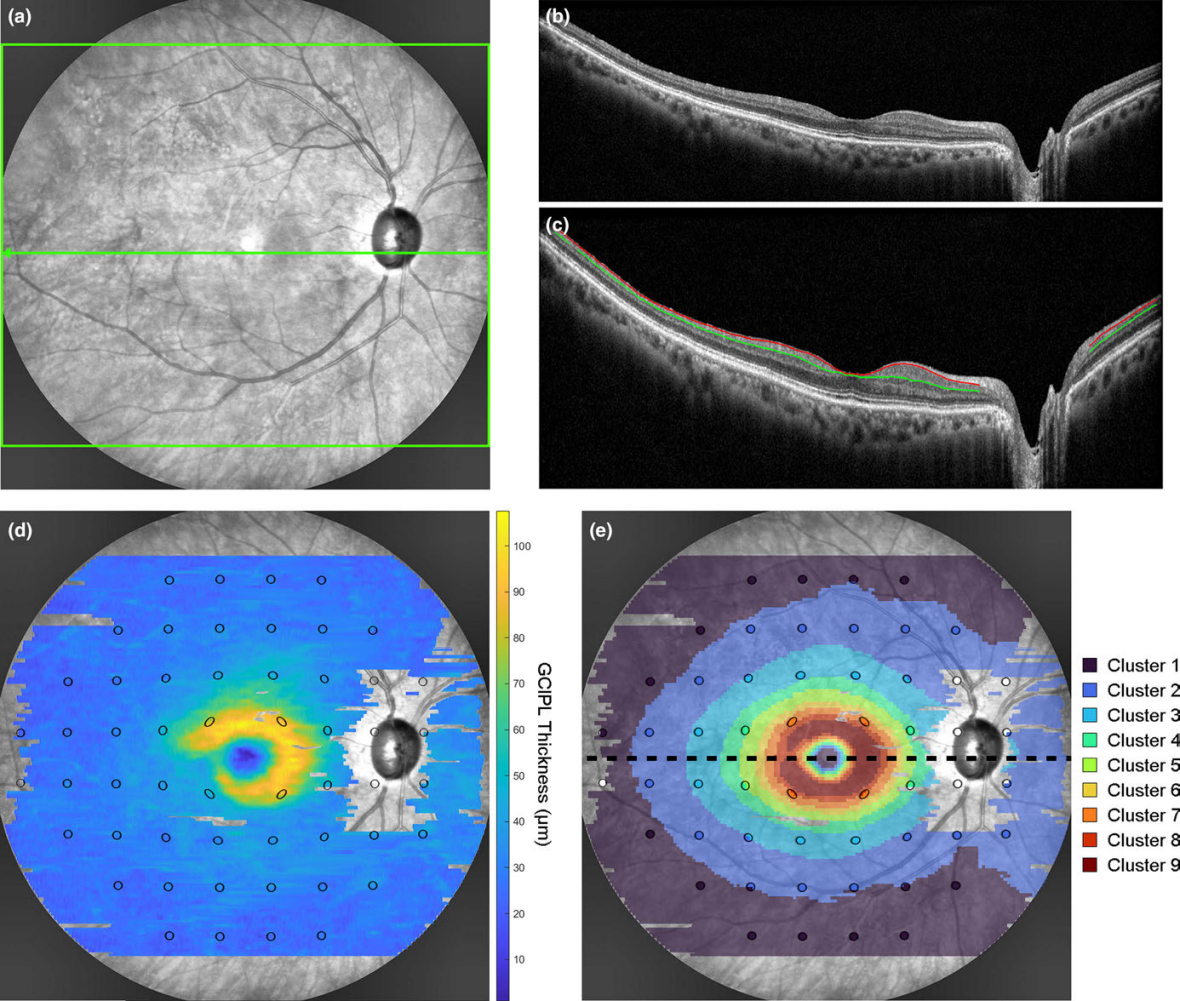
Example depicting the acquisition and processing of a widefield optical coherence tomography (OCT) scan, with all panels from a single glaucomatous eye. (a) The scanning laser ophthalmoscopy image with the scan area of the widefield OCT scan (green box) and location of the horizontal foveal B-scan (green arrow). (b) The corresponding raw foveal B-scan from A. (c) The same foveal B-scan from B. with warpage correction applied and segmentation of the retinal nerve fibre layer-ganglion cell layer (red line) and inner plexiform layer-inner nuclear layer boundaries (green line) to derive the ganglion cell-inner plexiform layer (GCIPL). (d) The GCIPL thickness map across the widefield OCT scan area, with 24-2 visual field (VF) test locations (black ellipses) superimposed. Where the scanning laser ophthalmoscopy image is completely visible indicates retinal locations not included for further analyses due to poor segmentation that could not be reliably interpolated from neighbouring locations. (e) The corresponding cluster arrangement describing the spatial pattern of age-related change in GCIPL thickness as per Tong et al.^9^ Projected 24-2 VF test locations (black ellipses) tended to fall within five clusters, with varying assignments of paracentral locations to Cluster 4 or 5 and central locations to Cluster 6, 7 or 8. For percentile and principal components analyses, clusters were split into hemi-clusters along the horizontal midline (black dashed line).

After the above selection process, one widefield OCT volume scan was acquired per included eye using the Widefield Imaging Module on Spectralis OCT, and GCIPL thickness data from these scans were used in comparison to 24-2 VF results in subsequent analyses. The widefield OCT data acquisition and processing protocol have been described in detail previously.^9,14^ In brief, each volume scan consisted of 109 horizontal B-scans spaced 120 μm apart, to capture a total retinal area of 55° horizontally × 45° vertically in a single scan (Figure 1). This differs from previously described montaged OCT protocols, where several standard posterior pole OCT scans were acquired in different directions of gaze and stitched together to generate composite OCT data over an equivalent retinal area.^14,29,30^ Scans that did not meet a minimal overall signal strength of 15 dB were excluded. Keratometry and axial length (AL) measurements were also obtained using the IOL Master (Carl Zeiss Meditec Inc., zeiss.com/corporate/en/home.html) to enable adjustment for transverse magnification effects.^31^

Due to the custom nature of the OCT acquisition protocol, automated segmentation within the instrument review software was not available, necessitating the development of software enabling automated segmentation of the GCIPL. OCT B-scans were exported as image (.TIFF) files and input into a typical encoder–decoder neural network (U-Net),^9,32,33^ resulting in the classification of pixels as either anterior to the RNFL-GCL boundary, between the RNFL-GCL and inner plexiform layer (IPL)–inner nuclear layer (INL) boundaries and posterior to the IPL–INL boundary. A subsequent graph search was used to infer the initial RNFL-GCL and IPL-INL boundaries, which were manually inspected to verify boundary accuracy and corrected as required (Figure 1c). Both manual delineation and automated identification of blood vessel locations using the corresponding scanning laser ophthalmology image were performed to initially exclude regions with segmentation errors. GCIPL thickness measurements were derived from the RNFL-GCL and IPL-INL boundaries, with further processing performed using software coded in MATLAB Version 2023a (Mathworks, au.mathworks.com/) to correct for review software-induced curvature warpage, retinal tilt and axial length (Figure 1d, details provided in Supporting Information S1).^34–36^

As per Centre for Eye Health protocols,^28,37,38^ two reliable 24-2 VF results from the same clinical visit were available for the included eye in some participants; in these cases, the second performed VF was selected for comparison to structural data. For each participant, mean deviation, pattern standard deviation, pointwise threshold, total deviation and pattern deviation data and corresponding probability maps were extracted. Hodapp-Parrish-Anderson criteria, defining a significant VF defect as falling within a group of at least three contiguous points flagged as *p* < 5% with at least one of these flagged as *p* < 1%, were applied to pattern deviation probability data to classify VF locations as defective or non-defective in glaucoma and glaucoma suspect eyes^26^; in eyes where the pattern deviation probability map was unavailable due to a severely depressed hill of vision, all VF locations were classified as defective. VF data were also used to classify glaucoma participants as pre-perimetric, early, moderate or advanced, using established classification criteria.^4^ Additionally, participants were classified as having advanced glaucoma when there was historical 10-2 VF damage at a concordant location to central or paracentral 24-2 VF defects.^39,40^

#### Quantitative relationships between estimated GC counts and VF thresholds

First, this study sought to characterise quantitative relationships between pointwise GC counts estimated from widefield OCT-derived GCIPL thickness measurements and 24-2 VF threshold data. Averaged GCIPL thicknesses were extracted over 24-2 test locations projected onto widefield GCIPL thickness maps using a Goldmann III stimulus size (0.43°) and a 0.5° allowance for microsaccades (Figure 1d).^41–43^ Projected stimulus locations and diameters were converted from visual angle to millimetres using corrections accounting for magnification variations across the tangential plane.^44,45^ Additionally, to account for lateral displacement of GCs from connecting photoreceptors within the maximum displacement zone,^46^ the margins of the central 16 24-2 locations were displaced according to Henle fibre length.^18,46^ Averaged GCIPL thicknesses were converted to GCL thicknesses using the equation described in Tong et al.^18^:

$$ \mathrm{GCL}=0.5651\times \mathrm{GCIPL}-0.9026 $$

GCL thicknesses were subsequently multiplied by co-localised GC densities calculated from histological human GC density data,^17^ to derive estimated GC counts for each participant and VF stimulus location (details available in Supporting Information S2).^16,47–49^

VF thresholds were plotted as a function of estimated GC counts, with data stratified according to diagnostic category (healthy, glaucoma suspect or glaucoma). Data were also stratified by VF defective or non-defective status, due to known high variability within VF defective locations particularly beneath the pseudo-measurement floor.^50,51^

#### Percentile-based method for comparing GCIPL and VF data

Next, for comparability to clinical assessments of structure–function concordance, agreement between metrics derived from GCIPL thickness measurements and VF data binarised to within or outside of normative limits was assessed. While an accurate pointwise approach would be valuable in identifying location-specific variations in the structure–function relationship, previous studies have reported high variability and poor predictability when applied to widefield and macular approaches.^30,47,52^ Therefore, both GCIPL and VF data were pooled across clusters as per Tong et al.^9^ (Figure 1e). This approach shares similar overarching concepts to comparisons of structure and function using the peripapillary RNFL using the Garway-Heath map,^12,13,53–55^ but using clusters derived from widefield GCIPL thickness analyses rather than being inferred from the RNFL. Projected 24-2 VF locations tended to fall within five clusters, with varying assignments of paracentral locations to Cluster 4 or 5 and central locations to Cluster 6, 7 or 8 due to varying fovea to optic disc tilts; as such, these clusters were grouped together for further analysis. Additionally, given the predisposition of glaucomatous defects to respect the horizontal midline,^56–59^ the cluster map was divided along the horizontal meridian to produce superior and inferior hemi-clusters.

**FIGURE 2:**
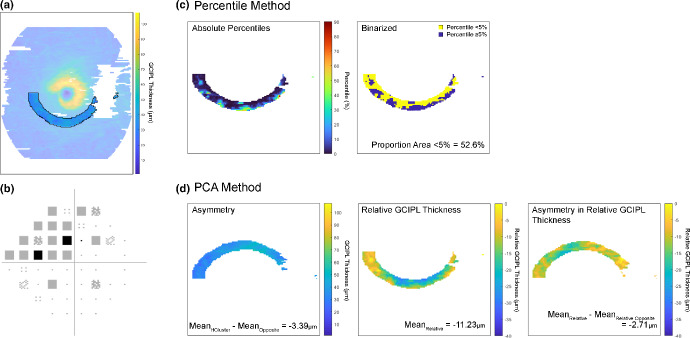
Schematic describing analysis methods to produce ganglion cell-inner plexiform layer (GCIPL)-based methods, for the same eye depicted in Figure 1, in the hemi-cluster corresponding to Cluster 3 and the superior visual field (VF) hemifield, or the inferior retinal hemifield. (a) The GCIPL locations corresponding to the hemi-cluster. (b) The pattern deviation map from 24-2 VF results, with the test locations corresponding to the hemi-cluster in A in bold. (c) The percentile method, where each individual measurement was compared to the distribution of measurements at the same location from the normative database, and the proportion of the area falling beneath the 5th percentile was calculated. (d) The principal components analysis (PCA) method, where the mean (mean_HCluster_) and standard deviation GCIPL thickness across the hemi-cluster in A, the difference relative to the mean GCIPL thickness across the other hemifield (asymmetry), the mean relative GCIPL thickness and asymmetry in relative GCIPL thickness were condensed into at least one principal component.

Individual GCIPL thickness data were compared to the Tong et al.^9^ normative database to derive OCT-based classifications of defective versus non-defective, akin to deviation map analyses in commercial OCT instrument review software (Figure 2a). For each participant, GCIPL thicknesses were averaged across grid squares measuring 100 × 100 μm over a total grid area of 160 × 160 grid squares, matching the resolution in Tong et al.^9^ Then, after matching for participant age and fovea to optic disc tilt, each grid square GCIPL measurement was compared to the distribution of normative database measurements for the corresponding grid square. Should the participant's measurement fall outside of the 5th percentile of the normative database distribution, that grid square would be flagged as defective, consistent with other OCT studies and commercial OCT software as a threshold for abnormality.^23^ The proportion of GCIPL locations flagged as defective were identified per hemi-cluster.

VF data were also grouped by hemi-cluster to match GCIPL data and classified binarily as VF defective or non-defective. Given the smallest, central hemi-clusters consisted of only two VF data points, a maximum classification criterion would be 50% of points within a hemi-cluster as VF defective. However, due to the increasing hemi-cluster size and therefore number of VF locations with increasing eccentricity, this criterion would classify peripheral hemi-clusters as non-defective despite a reasonable proportion of the hemi-cluster including defective data points. To balance the requirements of central and peripheral hemi-clusters, the criterion to classify hemi-clusters as VF defective or non-defective was set at 25%.

#### Principal components analysis method for comparing GCIPL and VF data

A potential pitfall of the above percentile-based method is that it does not reflect data variability within a hemi-cluster and GCIPL thicknesses relative to other retinal locations. To address these while limiting the complexity of including numerous potentially inter-correlated GCIPL thickness parameters, principal components analyses (PCA) were performed using GraphPad Prism Version 8.4.3 (graphpad.com) to condense the following GCIPL thickness parameters into at least one principal component (PC) for subsequent comparison to binarised VF data (Figure 2b):

1. Mean GCIPL thickness across each hemi-cluster.
2. Standard deviation of GCIPL thickness across each hemi-cluster.
3. Asymmetry in GCIPL thickness, calculated by subtracting the mean GCIPL thickness from the corresponding hemi-cluster in the opposite hemifield from 1.
4. Mean relative GCIPL thickness across each hemi-cluster.
5. Asymmetry in relative GCIPL thickness, calculated as per 3.

For steps 4 and 5, listed above, a relative GCIPL thickness map was generated for each participant using a process inspired by the pattern deviation concept.^60^ The absolute change relative to the mean normative database GCIPL measurement was calculated for each grid square, and the 85th percentile of this ‘total deviation’ of GCIPL thickness was set to zero. The relative GCIPL thickness map was generated from differences between all other locations and the 85th percentile, consistent with pattern deviation analyses.^60^

To ensure that the data were not over-fitted, PC regression between PCs derived from the five parameters and VF defect classifications per hemi-cluster were performed. Where non-significant parameters were observed, a process akin to backwards stepwise elimination was performed, where the least significant parameter was excluded, and PC regression repeated until all parameters were significant. These parameters and their corresponding eigenvectors were utilised in the final PCs (Tables S1 and S2).

### Statistical analysis

Additional statistical analyses were conducted using GraphPad Prism Version 8.4.3 and R Version 4.3.0 (R Foundation for Statistical Computing, r-project.org/). To characterise the quantitative relationships between pointwise GC counts estimated from widefield OCT-derived GCIPL thickness measurements and 24-2 VF threshold data, segmented linear regressions were applied with an initial breakpoint estimate of 15.5 dB, in line with established models using histologically derived GC counts.^21,22^ For each diagnostic category and VF defective or non-defective status, *F* test comparisons to linear regression models and Davies' tests were applied to identify the appropriateness of segmented regression models. To identify whether a single model was sufficient, *F* tests were also conducted between individual regression models for each of the five categories and a single regression model fitted to all the data. This was repeated across all data classified as VF non-defective and VF defective, to determine similarly whether different structure–function relationships existed across diagnostic categories. Mean absolute error and root mean square error were also calculated for each segmented linear regression model.^30,61,62^

For the percentile and PCA methods, agreement with VF data binarised as defective or non-defective was compared by calculating the areas under the receiver operating characteristic curve (AUROCCs). As analyses were intended to determine thresholds for optimal sensitivities and specificities based on the generated receiver operating characteristic curves (ROCCs), AUROCCs were calculated with data from a subset of 119 normal and 90 perimetric glaucoma participants, hereby termed the ROCC cohort, to avoid subsequent overfitting when applied to the entire cohort. For the percentile method, proportions of GCIPL locations flagged as beneath the 5th percentile were stratified by VF defective or non-defective status. For PCA methods, this involved simple logistic regression when there was one significant PC and multiple logistic regression when there was more than one significant PC. Additionally, the process of PCA with subsequent logistic regression was repeated with predicted outcomes for more central clusters based on logistic regression models, due to the likely overlap of glaucomatous VF defects across clusters within the same hemifield.^56,59^ For example, for Clusters 4–5, the predicted outcomes for the relatively central Clusters 6–8 were included as an additional parameter in PCA, and the outcomes of PC regression and corresponding eigenvectors were utilised in these PCs (Tables S3 and S4). The AUROCCs for the PCA and PCA plus central methods were compared with those from the percentile method using the DeLong test.^63^

The percentile method was chosen in the absence of significant differences in AUROCCs or the more complex PCA or PCA plus central method was chosen should the AUROCC significantly improve. The chosen methods per hemi-cluster were applied to the entire cohort. For each hemi-cluster, thresholds for optimal sensitivity and specificity were derived from the ROCC cohort using Youden's criterion, the maximum sum of sensitivity and specificity,^64^ and sensitivities and specificities with corresponding 95% confidence intervals were calculated for the ROCC cohort and the remaining cohort, hereby referred to as the test cohort, per the methods described by Ying et al.^65^ Throughout this study, the level of significance was set at *p* < 0.05.

## RESULTS

### Participant demographics

A total of 848 participants were included in this study, consisting of 311 healthy, 268 glaucoma suspect and 269 glaucoma participants (Table 1). Of the glaucoma participants, 88 were classified as pre-perimetric, 90 as perimetric and early, 41 as moderate and 50 as advanced. Across all participants regardless of diagnostic category, the axial length range was 21.03–27.60 mm. The details of the ROCC cohort, consisting of 119 healthy and 90 perimetric glaucoma participants, are provided separately.

**TABLE 1:** Demographic characteristics of the study cohort and the receiver operating characteristic curve (ROCC) cohort.

Diagnostic category	N	Age (y ± SD)	Sex (M:F)	SE (D ± SD)	VA (logMAR ± SD)	IOP (mmHg ± SD)	AL (mm ± SD)	Eye (OD:OS)	Tilt (° ± SD)	MD (dB ± SD)	PSD (dB ± SD)
Healthy	311	54.8 ± 14.6	150:161	−0.37 ± 1.92	0.03 ± 0.08	14.77 ± 2.96	23.97 ± 1.16	170:141	6.73 ± 3.59	−0.65 ± 1.26	1.65 ± 0.79
Glaucoma Suspect	268	62.2 ± 12.5	153:115	−0.14 ± 1.98	0.05 ± 0.09	15.96 ± 3.33	23.95 ± 1.11	135:131	6.65 ± 4.27	−1.16 ± 2.00	2.04 ± 1.31
Glaucoma	269	65.3 ± 11.3	163:104	−0.67 ± 2.11	0.07 ± 0.11	14.65 ± 3.93	24.18 ± 1.18	108:161	6.98 ± 3.94	−4.17 ± 5.20	4.33 ± 3.41
Pre-perimetric	88	66.8 ± 9.3	45:43	−0.33 ± 2.11	0.06 ± 0.08	14.75 ± 3.07	24.2 ± 1.09	43:45	6.91 ± 3.68	−1.00 ± 1.82	1.86 ± 1.05
Early	90	63.8 ± 10.8	60:30	−0.85 ± 2.14	0.06 ± 0.08	14.62 ± 3.81	24.04 ± 1.12	23:67	7.18 ± 4.09	−2.36 ± 1.65	3.16 ± 1.56
Moderate	41	62.9 ± 14.8	30:11	−0.96 ± 2.59	0.07 ± 0.09	14.44 ± 5.14	24.32 ± 1.46	20:21	6.9 ± 4.18	−7.69 ± 2.78	7.44 ± 3.00
Advanced	50	67.5 ± 11.4	30:20	−0.69 ± 2.12	0.11 ± 0.16	14.7 ± 5.95	24.28 ± 1.19	22:28	6.79 ± 4.02	−10.13 ± 7.74	8.21 ± 3.58
ROCC Cohort											
Healthy	119	59.2 ± 12.7	63:56	−0.26 ± 1.77	0.04 ± 0.09	14.09 ± 3.32	23.99 ± 1.15	61:58	6.45 ± 3.37	−0.55 ± 1.04	1.59 ± 0.54
Glaucoma	90	63.3 ± 11.8	59:31	−0.6 ± 2.17	0.08 ± 0.13	14.91 ± 5.18	24.17 ± 1.28	35:55	7.19 ± 3.43	−6.88 ± 5.98	6.18 ± 3.44

*Note*: Continuous values are shown as mean ± standard deviation (SD) where applicable.

Abbreviations: °, degrees; AL, axial length; D, dioptres; dB, decibels; F, female; IOP, intraocular pressure; logMAR, logarithm of the minimal angle of resolution; M, male; MD, mean deviation; mm, millimetres; mmHg, millimetres of mercury; N, number of participants; OD, right eye; OS, left eye; PSD, pattern standard deviation; SE, spherical equivalent refractive error; VA, visual acuity; y, years.

### Relationship between estimated GC counts and VF thresholds

**FIGURE 3:**
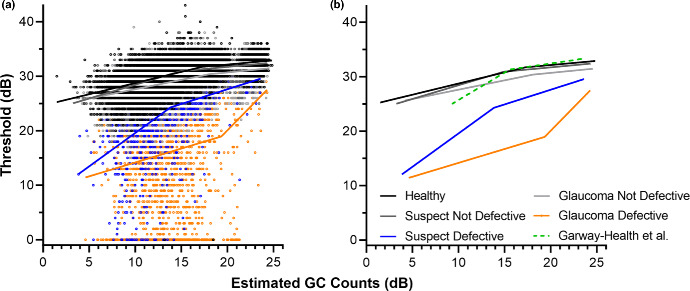
(a) Relationships between estimated ganglion cell (GC) count over GIII stimulus areas and 24-2 visual field (VF) thresholds, with data stratified by diagnostic category and VF defective/non-defective status. (b) The segmented linear regression models shown in isolation, with the Garway-Heath et al.^21^ superimposed.

Analyses of regression models describing the quantitative relationships between widefield OCT-derived GC counts and VF thresholds revealed that segmented linear models represented the data significantly better than linear models, irrespective of diagnostic category or VF status (*p* < 0.0001 to 0.02, Table 2 and Figure 3). Individual models for each diagnostic category and VF status demonstrated superior fits to a model fit to the overall data (*F* = 10698.84, *p* < 0.0001), with the VF defective regression models overall demonstrating much steeper slopes and smaller intercepts. Interestingly, while this tendency for a superior fit with individual models was maintained when comparing VF non-defective data across the different diagnostic categories (*F* = 667.89, *p* < 0.0001), there was a maximum predicted difference of 1.67 dB using individual models across the measured GC count range, suggesting that, at non-defective locations, there may be little practical benefit in using specific regression models per diagnostic category. Direct comparison of the VF non-defective data with the Garway-Heath model^21^ revealed overall consistent Slope1's to the left of the breakpoint but shallower Slope2's to the right of the breakpoint, reflecting discrepancies at more peripheral locations. While significant differences in regression models describing VF defective locations were also observed (*F* = 98.76, *p* < 0.0001), the maximum predicted difference between suspect and glaucoma models was 8.14 dB, suggesting greater discrepancies between diagnostic categories. Moreover, the larger variability in VF defective data, indicated by the low adjusted *R*^2^ values (0.04–0.21) and larger mean absolute and root mean square errors (4.40–8.89 dB and 6.10–9.93 dB, respectively) suggests poor predictive value of these models, especially in glaucoma eyes.

**TABLE 2:** Parameters of segmented linear regression for each diagnostic category and visual field defective or non-defective status.

	Healthy	Suspect—No defect	Suspect—Defect	Glaucoma—No defect	Glaucoma—Defect	No defect overall	Defect overall
Intercept	24.65	23.39	7.34	24.19	9.08	24.26	10.85
Slope1	0.41	0.5	1.22	0.34	0.51	0.41	0.51
Breakpoint	17.26	14.9	13.84	18.02	19.35	17.8	17.31
*p*-value	<0.0001	<0.0001	0.01	<0.0001	0.009	<0.0001	0.04
Slope2	0.15	0.16	0.54	0.17	1.73	0.14	1.00
Adjusted *R*^2^	0.32	0.31	0.21	0.18	0.04	0.26	0.04
*F*	137.01	198.59	5.87	17.86	4.18	256.67	2.64
*p*-value	<0.0001	<0.0001	0.003	<0.0001	0.02e	<0.0001	0.07
MAE (dB)	1.43	1.59	4.40	1.91	8.89	1.66	8.11
RMSE (dB)	1.89	2.06	6.10	2.51	9.93	2.20	9.58

*Note*: *p*-Values beneath breakpoints indicate results of Davies' tests. *F* statistics and corresponding *p*-values indicate comparisons between segmented linear and linear regression models.

Abbreviations: dB, decibels; MAE, mean absolute error; *R*^2^, coefficient of determination; RMSE, root mean square error.

### Classification of VF status using GCIPL-based methods

**FIGURE 4:**
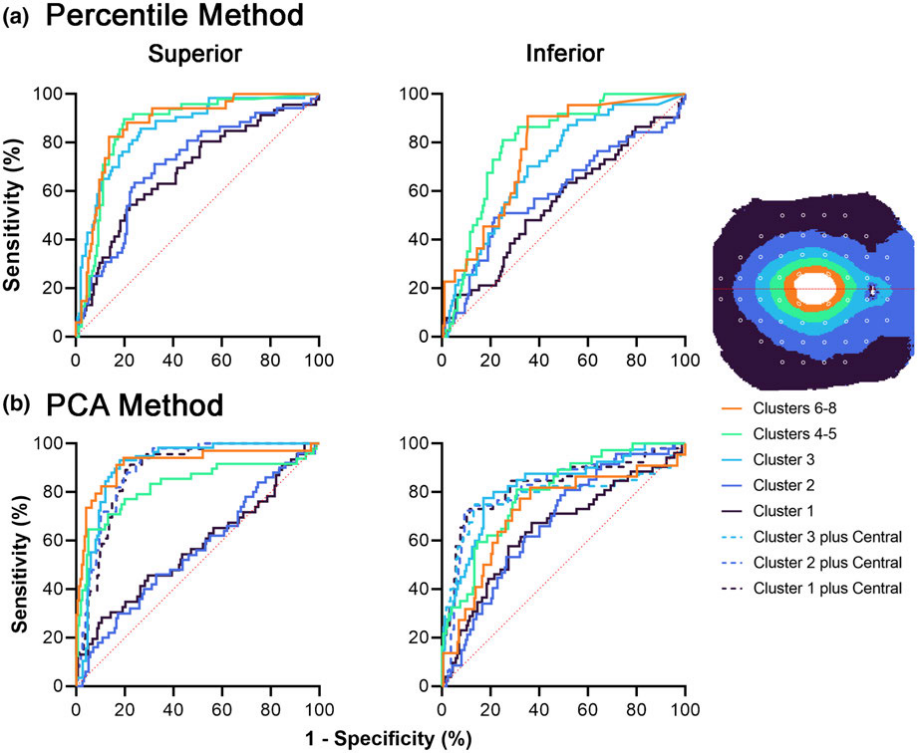
Receiver operating characteristic curves (ROCCs) per hemifield for (a) the percentile and (b) principal components analysis (PCA) methods used in binary classification into visual field defective versus non-defective locations from ganglion cell-inner plexiform layer thicknesses, with the line of no discrimination included (red dotted line). Note in (b), ROCCs at Clusters 1–2 improved when including the predicted VF statuses for relatively central locations (dashed lines). The corresponding locations per hemi-cluster are provided in the legend, with the red line indicating the horizontal midline from which superior and inferior hemifields were separated. Per convention, superior and inferior hemifields refer to the visual field format.

Using widefield OCT-derived GCIPL thickness parameters and binarised VF classifications as defective or non-defective, AUROCC comparisons within the ROCC cohort between the percentile, PCA and PCA plus central hemi-cluster classifications methods revealed no significant differences in the central two clusters in both hemifields (Table 3 and Figure 4). Meanwhile, the PCA method demonstrated significantly higher AUROCCs than the percentile method in Cluster 3, with no further improvement using the PCA plus central method. The AUROCCs in Clusters 1 and 2 were significantly larger using the PCA plus central method across both hemifields, indicating that the inclusion of the predicted VF status for more central locations significantly improved performance in these relatively peripheral clusters. Using the most appropriate GCIPL-based method from these comparisons, 84.0% (*n* = 152) of participants with perimetric glaucoma demonstrated at least one hemi-cluster of corresponding abnormal GCIPL and VF results, which is comparable to previous methods.^23^ Meanwhile, using pointwise GCIPL data or data directly corresponding to retinal locations stimulated during VF assessment only, significantly poorer AUROCCs for Cluster 1 in the superior hemifield and Cluster 2 across both hemifields were observed, indicating poorer discrimination at relatively peripheral locations using pointwise data (Supporting Information S3).

**TABLE 3:** Areas under the receiver operating characteristic curves (AUROCCs) for each of the methods used in binary classification into visual field defective versus non-defective locations from ganglion cell-inner plexiform layer thicknesses.

	1. Percentile	2. PCA	*p*-Value, 1 versus 2	2b. PCA plus central	*p*-Value, 1 versus 2b
Superior hemifield					
Clusters 6–8 (central)	**0.87 (0.030)**	0.92 (0.032)	0.06		
Clusters 4–5	**0.86 (0.029)**	0.83 (0.041)	0.31	0.86 (0.038)	0.84
Cluster 3	0.86 (0.028)	**0.91 (0.021)**	0.0003	0.9 (0.022)	0.001
Cluster 2	0.71 (0.042)	0.56 (0.047)	0.04	**0.9 (0.022)**	<0.0001
Cluster 1 (peripheral)	0.68 (0.046)	0.57 (0.051)	0.01	**0.88 (0.024)**	<0.0001
Inferior hemifield					
Clusters 6–8 (central)	**0.77 (0.046)**	0.72 (0.064)	0.29		
Clusters 4–5	**0.8 (0.035)**	0.80 (0.038)	0.97	0.81 (0.034)	0.71
Cluster 3	0.71 (0.041)	**0.82 (0.039)**	0.0001	0.78 (0.053)	0.05
Cluster 2	0.59 (0.049)	0.67 (0.042)	0.14	**0.82 (0.038)**	<0.0001
Cluster 1 (peripheral)	0.55 (0.047)	0.65 (0.046)	0.05	**0.83 (0.038)**	<0.0001

*Note*: Per convention, superior and inferior hemifields refer to the visual field format. Standard errors of the mean are included in brackets. AUROCCs highlighted in bold indicate the corresponding method chosen for subsequent analyses, either due to being the simpler method in the absence of significant differences or the more complex method with a significant improvement in AUROCC.

Abbreviations: PCA, principal components analysis.

From thresholds based on Youden's criterion applied to the ROCC cohort (Table S5), sensitivities and specificities remained reasonably consistent across all hemi-clusters within the ROCC cohort and test cohort, which was naïve to previous analyses (Table 4), with no drop-off in sensitivity and specificity at relatively peripheral locations. Across all hemi-clusters, while no significant differences in specificity between the ROCC and test cohorts were observed, significantly lower sensitivities were observed in the test cohort. This difference appeared due to the addition of glaucoma suspect eyes in the test cohort, supported by the poorer observed sensitivities in the glaucoma suspects when using calculations stratified by diagnostic category (Table S6). In analyses stratified by diagnostic category, specificities were overall highest in healthy and glaucoma suspect eyes (Table S7); the low specificities across glaucoma eyes indicate a propensity for false positives, or incorrect identification of VF defective locations given GCIPL measurements outside of normative limits. Due to the apparent discrepancies in the glaucoma suspect cohort, analyses were repeated on a subset of 190 eyes with two reliable VF results from the same day available with an additional criterion of the VF defect being repeatable across both attempts. This subanalysis revealed similar sensitivities but improvements in specificities across all hemi-clusters (Table S8).

**TABLE 4:** Sensitivities and specificities with 95% confidence intervals calculated using the optimal ganglion cell-inner plexiform layer (GCIPL)-based method in the receiver operating characteristic curve (ROCC) and test cohorts.

	Sensitivity		Specificity	
	ROCC cohort	Test cohort	ROCC cohort	Test cohort
Superior hemifield				
Clusters 6–8 (central)	0.82 (0.7–0.95)	0.53 (0.36–0.70)	0.86 (0.81–0.91)	0.89 (0.86–0.91)
Clusters 4–5	0.90 (0.81–0.98)	0.63 (0.52–0.74)	0.80 (0.74–0.86)	0.80 (0.77–0.83)
Cluster 3	0.86 (0.77–0.94)	0.60 (0.5–0.70)	0.73 (0.66–0.80)	0.62 (0.58–0.66)
Cluster 2	0.86 (0.76–0.96)	0.55 (0.43–0.66)	0.81 (0.74–0.87)	0.81 (0.78–0.84)
Cluster 1 (peripheral)	0.91 (0.83–0.99)	0.60 (0.47–0.72)	0.78 (0.72–0.84)	0.80 (0.76–0.83)
Inferior hemifield				
Clusters 6–8 (central)	0.91 (0.79–1.00)	0.58 (0.42–0.74)	0.72 (0.65–0.78)	0.65 (0.61–0.69)
Clusters 4–5	0.81 (0.68–0.94)	0.36 (0.25–0.47)	0.77 (0.71–0.84)	0.74 (0.70–0.78)
Cluster 3	0.93 (0.87–1.00)	0.68 (0.58–0.77)	0.82 (0.76–0.88)	0.72 (0.69–0.76)
Cluster 2	0.72 (0.60–0.85)	0.47 (0.36–0.58)	0.91 (0.86–0.95)	0.87 (0.84–0.90)
Cluster 1 (peripheral)	0.73 (0.61–0.85)	0.47 (0.35–0.58)	0.89 (0.84–0.94)	0.84 (0.81–0.87)

*Note*: Per convention, superior and inferior hemifields refer to the visual field format.

## DISCUSSION

The present study utilised GCIPL parameters derived from widefield OCT volume scans spanning the retinal area sampled during 24-2 VF assessment, and explored their relationships with quantitative and binarised VF data across a spectrum of healthy, glaucoma suspect and glaucoma eyes. While the segmented linear models describing the relationships between GC counts and VF thresholds were comparable to the established Garway-Health et al.^21^ model at non-defective locations, the greater variability at VF defective locations suggests these may not be useful in predicting VF thresholds from widefield OCT. Comparisons between binarised VF results and GCIPL thickness-derived factors revealed low sensitivities in the glaucoma suspect group and low specificities in the glaucoma cohorts, indicating the persistence of structure–function discordance using widefield OCT. While these results suggest that accurate prediction of VF results may not be possible using the widefield OCT alone, the overall reasonable specificities across all diagnostic categories and high specificities in healthy participants suggest that this approach may be useful to screen for eyes that would benefit from additional VF testing.

### Comparisons with prior studies

The validity of the widefield OCT approach in predicting GC counts from GCIPL thickness measurements was tested via comparison to the established Garway-Health et al.^21^ model. The relatively shallow Slope2's of the widefield OCT regression models at non-defective locations is likely an artefact of the different VF algorithms used between studies; while direct comparisons of SITA Faster and Full Threshold strategies are unavailable, higher SITA Faster thresholds have been reported in conjunction with lower SITA Standard thresholds,^28,66^ and similar algorithm-specific trends have not been reported between SITA Standard and Full Threshold.^67,68^ As test–retest variabilities between SITA Faster and SITA Standard appear quite similar,^37,69^ these findings could reflect some differences in the dynamic range and number of steps in SITA Faster relative to older VF algorithms. Further investigations of these properties of SITA Faster would be valuable given the implications for ongoing monitoring when switching between algorithms.

The large range of VF thresholds corresponding to each GC count, especially at VF defective locations, could indicate either some error in GC counts estimated from GCIPL thickness alone, inherent variability in VF data^37,51,70^ or both. In glaucomatous eyes, decreases in GCIPL thickness may be observed in conjunction with changes in GC density along the retinal plane^71,72^ or alterations to GC soma size.^73–75^ Additionally, while segmentation errors induced by the larger intraretinal vessels were corrected in the processing stage, reductions in inner retinal capillary density and morphological changes to Müller cells and astrocytes have been reported in glaucoma,^76–78^ and given their contribution to GCIPL thickness, variations in these non-neural components may confound estimates of GC counts from OCT in glaucomatous eyes. However, variations in GC densities across the retina in glaucomatous eyes have not been reported to the level of detail by Curcio and Allen,^17^ which would indeed be valuable in potentially producing more accurate GC count estimates in disease. Moreover, known inter- and intra-individual variability certainly highlights the imperfection of using VF data as the standard of detective status, although assessment of visual function remains imperative in glaucoma due to associations with quality of life and ongoing use as a clinical trial endpoint.^79^ Performing multiple VF assessments at a single visit may be a viable method to limit intra-individual variability,^37^ and the improvement in specificity when using repeated VF data in the glaucoma suspect cohort highlights the potential benefits of repeated VF testing in clinical assessments of structure–function concordance. Previous work has suggested that floor-related effects would hamper the ability of the GCIPL to predict VF status at the peripheral macula and beyond,^52^ equivalent to Cluster 3 in this study. However, reasonable correlations between histologically measured GC density and GCIPL thickness measured using OCT have been reported out to 12° eccentricity in primate eyes,^71^ and changes to GC soma and dendritic tree area in glaucoma have been reported previously.^73,74,80,81^ Given that GC somas and dendrites correspond to the GCL and IPL, respectively, alterations in these structures due to increasing GC dysfunction in glaucoma could theoretically translate to changes in GCIPL thickness, even in non-macular regions. The relatively low AUROCCs at Clusters 1 and 2 using both percentile and PCA-based methods indicate that the thin GCIPL tissue in these regions confers a limited dynamic range affecting the ability of OCT to discriminate between healthy and diseased GCIPL. However, AUROCCs consistent with those at the central hemi-clusters were observed in Clusters 1 and 2 using the PCA plus central method, suggesting that the incorporation of predictions based on relatively central locations can improve discrimination between VF defective and non-defective status using widefield OCT across the entire measurement area.

While other widefield OCT studies included the RNFL in structural assessments and are therefore not directly comparable to this study, using a similar binarised approach to determine abnormal structure and function, Hood et al.^23^ reported that 88.7% of their glaucoma cohort demonstrated structure–function concordance, very similar to the 84.0% found in the present study. Studies using widefield and peripapillary RNFL to predict both global and pointwise 24-2 VF parameters reported mean absolute errors of 4.25–8.40 dB and root mean square errors of 3.30–5.90 dB^30,61,62^; interestingly, these fell between the error parameters generated from the no defect and defect segmented linear models in the present study, which may also imply that VF defective locations contribute most to the variability in these models.

### Apparent discordance in glaucoma suspects and glaucoma

The persistence of structure–function discordance, as demonstrated by the low sensitivities observed in the glaucoma suspect cohort and low specificities across glaucoma cohorts, suggests that factors in addition to acquisition of co-localised OCT and VF data contribute to this phenomenon, contrary to the study hypothesis. Despite attempts to control for inter-individual variability in retinal thickness via comparisons to normative data, variations in GC soma size and density between individuals and in disease may contribute to retinal thickness measurements being an imperfect surrogate of GC health, especially in the early stages of the disease. Investigations of mouse models of glaucoma reported lower densities of immunohistorochemical labelling of GC axons and mRNA for GC-specific genes, suggesting GC axonal dysfunction may occur prior to observable changes to GC soma density^75^; this observation appears consistent with the present study's findings of the potential for VF defects to be observed despite no apparent co-localised retinal thinning on OCT.

Perhaps, it is not surprising that reductions in GCIPL thickness were observed using widefield OCT in pre-perimetric glaucoma patients, given that a hallmark feature of pre-perimetric glaucoma is structural damage which may involve the macular GCIPL in the absence of corresponding VF damage.^82^ While discordance between GCIPL and VF findings could be attributed to the GIII stimulus being larger than Ricco's area centrally, with the resultant incomplete spatial summation masking GC dysfunction and corresponding functional outputs,^83–86^ this would not explain the apparent discordance at relatively peripheral locations where GIII and Ricco's area become more equivalent in size.^84^ This is unlikely to be related to peripheral defocus effects resulting in greater variability in boundary segmentation, given previously reported differences of 1.00 D between auto-refraction measurements acquired centrally and 30° from fixation,^87^ and that such small differences in focus have translated to minimal differences in Spectralis OCT-derived retinal thickness measurements.^88^ Additional analyses in the ROCC cohort also revealed only small differences in signal strength between foveal and peripheral B-scans (Supporting Information S4), and therefore, less reliable boundary segmentation at peripheral locations of the widefield OCT scan appears unlikely. An alternative possibility is that the apparent discordance could be related to the sparsity of test points in the 24-2 test grid, with glaucomatous structural defects perhaps falling in between VF test locations. Utilising a higher sampling density of test locations in regions of structural change could reveal corresponding functional defects.^89^ In this study, the generation of parameters across hemi-clusters per the GCIPL-based methods precluded such fine comparisons of widefield OCT and 24-2 VF results; however, widefield OCT approaches certainly offer an interesting opportunity to investigate the structure–function relationship in pre-perimetric glaucoma in the future.

### Future directions and potential clinical applications of widefield OCT

While the results of this study suggest that the widefield OCT protocol may be useful in identifying structure–function concordance across the glaucoma spectrum, several points of note would need to be addressed in future work to verify its clinical utility. First, while high intra-visit repeatability has been reported in primate widefield GCIPL thickness measurements,^15^ the repeatability of GCIPL findings acquired using the described widefield OCT protocol in humans would need to be established, in order to determine the extent of change falling outside of expected variability that may be pathological in nature. Second, a longitudinal study design monitoring patients with progressive disease would be highly informative; for example, should changes in GCIPL thickness concordant with VF progression be observed, then this would suggest more convincingly that these changes in widefield OCT-derived GCIPL measurements reflect structural changes to the underlying GCs, while an absence of change in GCIPL measurements may indicate that non-neuronal components confound measurements at peripheral locations.

Moreover, the GCIPL was originally chosen as the structural parameter of interest in this study, given the complexity surrounding considerations of inter-individual variations in RNFL trajectory which could impact comparisons to normative data. However, the relatively low sensitivities in the glaucoma suspect cohort, indicating the presence of VF defects with GCIPL metrics within normative limits, imply that the RNFL could be a useful structural parameter to consider in future studies of the widefield OCT. As the developed segmentation software only enabled the segmentation of the GCIPL, analyses using the RNFL were outside of the scope of this investigation. However, similar approaches to those described in this study, such as pooling data by RNFL clusters such as the Garway-Heath map,^53^ may be useful in future investigations of the widefield OCT.

With an expected increase in the prevalence of glaucoma in the context of an aging global population,^90^ accurate and efficient methods of identifying patients at risk of vision loss are required to limit the associated disease burden. While VF assessment remains the gold standard for assessing visual function, strategies to prudently identify eyes likely to demonstrate vision loss can ensure that the costs in performing additional testing are reserved for patients who would benefit most. The described GCIPL-based method to binarise locations as likely VF defective or non-defective may be useful for such a screening purpose, given the low false-positive rate suggested by the high specificity values in healthy and glaucoma suspect subcohorts. While the higher false-positive rates indicated by lower specificities in the glaucoma subcohorts are not ideal, performing VF assessments in these eyes to confirm the absence of corresponding vision loss is more appropriate, especially as these eyes demonstrated treatable structural damage using conventional assessments. In conjunction with its quick acquisition time, requiring less effort from the patient and practitioner perspective, widefield OCT approaches, such as that described in the present study, could aid clinical decision-making to ensure further VF assessments are targeted to appropriate patients.

### Limitations

In addition to the limitations described above, the demographic characteristics of patients attending the Centre for Eye Health and a single private ophthalmology practice may limit the generalisability of the study findings.^24,91^ First, the majority of glaucoma participants included in this investigation were classified as pre-perimetric or early, although sample sizes for moderate and advanced glaucoma patients were similar to previous studies.^30,61,62^ Second, due to a paucity of eyes with high refractive error in the normative database,^9^ eyes with a spherical equivalent outside +6.00 to −6.00 D were excluded from this study. Future work including a larger cohort of moderate to advanced glaucoma patients and/or eyes with high refractive error would be valuable to determine whether the present findings translate to these populations. Furthermore, a second reliable VF result was not available in all participants, due to a propensity for lower test reliabilities using SITA Faster,^28,38^ and a second VF result to ensure defect repeatability would have been valuable. Finally, the resolution of the widefield OCT protocol was insufficient to visualise the temporal raphe, and variations in GCIPL thickness related to temporal raphe position may have affected normative database comparisons.

### Conclusion

In this study, widefield OCT was utilised to obtain GCIPL data directly corresponding to 24-2 VF test locations, and the relationships between these GCIPL parameters and clinical VF data were explored across a spectrum of healthy, glaucoma suspect and glaucoma eyes. While quantitative relationships between estimated GC counts derived from widefield OCT and VF thresholds were largely consistent with previous studies at non-defective locations, quantitative prediction of VF thresholds from OCT would likely be affected by high variability at defective locations. A binarised approach of VF status based on GCIPL parameters, demonstrating high specificity in healthy eyes and reasonable specificity overall, could become useful to aid clinical decision-making in appropriately targeting VF assessments.

## Supplementary Information

Supplementary file (DOCX 56.2 KB)
