# Supplementary material for: Exploring the relationship between 24-2 visual field and widefield optical coherence tomography data across healthy, glaucoma suspect and glaucoma eyes
Source: Ophthalmic Physiol Opt. 2024 Jul 26;44(7):1484–99. doi: 10.1111/opo.13368 (PMC12891256; doi:10.1111/opo.13368)
Supplement: Supplementary file 1 — Supplementary file (DOCX 56.2 KB) [file 44402_2024_4407017_MOESM1_ESM.docx]

**Supplementary Materials for: Exploring the relationship between 24-2 visual field and widefield optical coherence tomography data across healthy, glaucoma suspect and glaucoma eyes**

Janelle Tong^1,2,3^, Jack Phu^1,2,3,4,5^, David Alonso-Caneiro^6,7^, Jason Kugelman^7^, Sieu Khuu^2^, Ashish Agar^8^, Minas Coroneo^8^, Michael Kalloniatis^2,3^

1. Center for Eye Health, University of New South Wales, Sydney, New South Wales, Australia
2. School of Optometry and Vision Science, University of New South Wales, Sydney, New South Wales, Australia
3. School of Medicine (Optometry), Deakin University, Waurn Ponds, Victoria, Australia
4. Faculty of Medicine, University of Sydney, Sydney, New South Wales, Australia
5. Concord Clinical School, Concord Repatriation General Hospital, Sydney, New South Wales, Australia
6. School of Science, Technology and Engineering, University of Sunshine Coast, Sunshine Coast, Queensland, Australia
7. Queensland University of Technology, Contact Lens and Visual Optics Laboratory, Centre for Vision and Eye Research, School of Optometry and Vision Science, Kelvin Grove, Queensland, Australia
8. Department of Ophthalmology, University of New South Wales at Prince of Wales Hospital, Sydney, Australia.

Running Title: Structure-Function with Widefield OCT

Supplementary Materials: 12 (4 methods, 8 tables)

**Supplementary Methods 1. Processing to Derive Ganglion Cell-Inner Plexiform Layer (GCIPL) Thickness Measurements**

Further processing performed using software coded in Matlab Version 2023a (Mathworks, Natick, MA USA) to correct for review software-induced curvature warpage, retinal tilt, and axial length.^1^ As optical coherence tomography (OCT) review software flattens B-scans at the periphery, warpage correction was conducted by readjusting boundaries to remain equidistant from the nodal point of the eye,^2-4^ the position of which was calculated from a combination of axial length and refractive error data.^5^ After the distances between corrected boundary locations were used to calculate axial GCIPL thickness (GCIPL_uncorrected_), these were further corrected to obtain GCIPL thicknesses perpendicular to the slope of the retina (GCIPL_tilt corrected­_) using the following equation:^1^

$$GCIPL_{tilt corrected}=GCIPL_{uncorrected}-14.03*tilt^{2}+1.611\times tilt-0.07437$$

Where tilt refers to retinal tilts relative to the foveal center in radians. Subsequently, corrected GCIPL thicknesses were linearly interpolated in areas previously excluded for segmentation errors, except at locations adjacent to the optic disc, within the macula and at the edges of the OCT scan, to generate corrected widefield GCIPL thickness maps. Finally, due to previously identified significant correlations between widefield-derived GCIPL thickness and AL independent of transverse magnification effects, GCIPL thicknesses were corrected to a standard AL of 24.385mm based on the coefficient derived from multiple linear regression:^1^

$GCIPL_{AL corrected}=GCIPL_{tilt corrected}-0.436\times(24.385-AL)$

**Supplementary Methods 2.** Derivation of Ganglion Cell (GC) Density Maps from Histological GC Data

Consistent with previously described methods,^6-8^ histological human GC density data reported in Curcio and Allen^9^ was used to generate a map of GC counts across the central 15.4mm of the retina at a resolution of 0.005mm/pixel. Then, estimated GCL thicknesses were calculated from widefield GCIPL thickness data from a previously described normative database of 470 eyes,^1^ which were age-corrected to match the mean age of the Curcio and Allen^9^ cohort, 34 years old. The GC count map was divided by averaged GCL thicknesses per pixel to derive an age-agnostic GC density per millimeter of GCL thickness map. Subsequently, this map was used to calculate GC densities averaged across projected 24-2 VF stimulus locations, accounting for individual eyes’ fovea to optic disc tilt and tangential magnification, and were multiplied by GCL thicknesses to derive GC counts for each participant and each VF stimulus location.

**Supplementary Methods 3. Pointwise Data and GCIPL Methods**

Further sub-analyses investigating ganglion cell-inner plexiform layer (GCIPL) methods using GCIPL data extracted across retinal locations corresponding to the 24-2 visual field (VF) test grid, or pointwise data, were performed as a comparison point to data extracted across hemi-clusters (Table 3 and Figure 4 in the main text). The percentile and principal components analysis (PCA) methods were applied to pointwise data. The PCA method involved collation of mean GCIPL thickness and mean relative GCIPL thickness across each pointwise location, as well as the difference with mean values in the opposite hemifield to derive asymmetry values for both; per methods described in the main text, principal components regression was used to include only significant parameters in the final PCA metrics. PCA with predicted outcomes based on logistic regression analyses for relatively central hemi-clusters was also performed, with the proportion of pointwise locations predicted to be VF defective utilized as the central hemi-cluster parameter.

AUROCCs for the PCA and PCA plus central methods were compared with those from the percentile method using the DeLong test (Supplementary Methods Table). Significantly higher AUROCCs were found using either the PCA method or the PCA plus central method for all but Cluster 4 in the superior hemifield; for consistency across hemi-clusters, PCA methods were used for further analyses for all hemi-clusters. Given significantly higher AUROCCs using the PCA plus central methods, these were chosen for Clusters 1 and 2 in the superior hemifield and Cluster 1 to 3 in the inferior hemifield. These were then compared to AUROCCs calculated from data extracted across hemi-clusters, revealing significant differences in AUROCCs for Cluster 1 in the superior hemifield and Cluster 2 across both hemifields. These results indicate significantly poorer discrimination between VF defective and non-defective locations using pointwise data at these relatively peripheral locations only.

***Supplementary Methods Table.*** *Areas under the receiver operating characteristic curves (AUROCCs) for each of the methods used in binary classification into visual field defective versus non-defective locations from pointwise (PW) ganglion cell-inner plexiform layer thicknesses. Per convention, superior and inferior hemifields refer to visual field format. Standard errors of the mean are included in brackets. AUROCCs highlighted in bold indicate the corresponding method chosen for comparison to AUROCCs derived from GCIPL data extracted across hemi-clusters (right-most column, hemi-cluster data can be found in Table 3 in the main text).*

|  | **1. Percentile** | **2. PCA** | **P value 1. vs. 2.** | **2b. PCA plus central** | **P value 1. vs. 2b.** | **P value PW vs. hemi-cluster** |
| --- | --- | --- | --- | --- | --- | --- |
| Superior hemifield | | | | | | |
| Clusters 6-8 (central) | 0.83 | **0.89** |  | 0.007 |  | 0.65 |
| Clusters 4-5 | 0.84 | **0.84** | 0.86 | 0.85 | 0.39 | 0.51 |
| Cluster 3 | 0.76 | **0.87** | 0.72 | <0.0001 | 0.09 | 0.16 |
| Cluster 2 | 0.59 | 0.61 | **0.84** | 0.40 | <0.0001 | 0.02 |
| Cluster 1 (peripheral) | 0.61 | 0.59 | **0.82** | 0.26 | <0.0001 | 0.02 |
| Inferior hemifield | | | | | | |
| Clusters 6-8 (central) | 0.69 | **0.82** |  | 0.002 |  | 0.36 |
| Clusters 4-5 | 0.68 | **0.77** | 0.77 | 0.0008 | 0.002 | 0.60 |
| Cluster 3 | 0.59 | 0.69 | **0.81** | 0.002 | <0.0001 | 0.82 |
| Cluster 2 | 0.56 | 0.59 | **0.72** | 0.06 | <0.0001 | 0.02 |
| Cluster 1 (peripheral) | 0.54 | 0.54 | **0.80** | 0.88 | <0.0001 | 0.46 |

**Supplementary Methods 4. Examining Differences in Signal Strength within the Widefield OCT Volume Scan**

Additional comparisons of B-scan quality scores for the fovea, superior-most and inferior-most B-scans for the receiver operating characteristic curve (ROCC) cohort were performed to investigate the possibility of peripheral defocus impacting B-scan signal strength, which may increase variability in the resultant retinal thickness measurements.^10, 11^ After the signal strength scores for the foveal, superior-most and inferior-most B-scan were extracted for each scan, repeated measures analysis of variance with adjustment for multiple comparisons was performed. There were no significant differences in B-scan quality between the foveal and superior-most B-scans (mean difference ± standard error of the mean of 0.14 ± 0.25, P = 0.567), but significant differences were noted between the foveal and inferior-most B-scans (mean difference ± standard error of the mean of 1.55 ± 0.14, P < 0.0001). However, quality scores across all locations were high, with mean and standard deviation quality scores of 32.34 ± 3.25 for the fovea, 32.20 ± 3.57 for the superior-most scans and 30.79 ± 4.34 for the inferior-most scans. That is, the reduction in signal strength at the inferior edge of the widefield OCT scans, while significant, is small, and unlikely to produce gross, systematic deviations in boundary segmentation.

**References for Supplementary Methods**

1. Tong J, Alonso-Caneiro D, Kugelman J, Phu J, Khuu SK & Kalloniatis M. Characterization of the normal human ganglion cell-inner plexiform layer using widefield optical coherence tomography. *Ophthalmic Physiol Opt*. 2024; 44 (2): 457-71.

2. Kuo AN, McNabb RP, Chiu SJ, et al. Correction of ocular shape in retinal optical coherence tomography and effect on current clinical measures. *Am J Ophthalmol*. 2013; 156 (2): 304-11.

3. McNabb RP, Polans J, Keller B, et al. Wide-field whole eye oct system with demonstration of quantitative retinal curvature estimation. *Biomed Opt Express*. 2019; 10 (1): 338-55.

4. Alonso-Caneiro D, Read SA, Vincent SJ, Collins MJ & Wojtkowski M. Tissue thickness calculation in ocular optical coherence tomography. *Biomed Opt Express*. 2016; 7 (2): 629-45.

5. Christaras D, Rozema JJ & Ginis H. Ocular axial length and straylight. *Ophthalmic Physiol Opt*. 2020; 40 (3): 316-22.

6. Raza AS & Hood DC. Evaluation of the structure-function relationship in glaucoma using a novel method for estimating the number of retinal ganglion cells in the human retina. *Invest Ophthalmol Vis Sci*. 2015; 56 (9): 5548-56.

7. Yoshioka N, Zangerl B, Phu J, et al. Consistency of structure-function correlation between spatially scaled visual field stimuli and in vivo oct ganglion cell counts. *Invest Ophthalmol Vis Sci*. 2018; 59 (5): 1693-703.

8. Tong J, Khou V, Trinh M, Alonso-Caneiro D, Zangerl B & Kalloniatis M. Derivation of human retinal cell densities using high-density, spatially-localized optical coherence tomography data from the human retina *J Comp Neurol*. 2023; 531 (11): 1108-25.

9. Curcio CA & Allen KA. Topography of ganglion cells in human retina *J Comp Neurol*. 1990; 300 (1): 5-25.

10. Cheung CY, Leung CK, Lin D, Pang CP & Lam DS. Relationship between retinal nerve fiber layer measurement and signal strength in optical coherence tomography. *Ophthalmology*. 2008; 115 (8): 1347-51, 51 e1-2.

11. Balasubramanian M, Bowd C, Vizzeri G, Weinreb RN & Zangwill L. Effect of image quality on tissue thickness measurements obtained with spectral-domain optical coherence tomography. *Opt Express*. 2009; 17 (5): 4019–36.

**Supplementary Table 1.** Parameter estimates from the original principal components regression with all variables included, with P values per parameter estimate in brackets. Per convention, superior and inferior hemifields refer to visual field format.

|  | **Intercept** | **Mean** | **SD** | **Asymmetry** | **Relative GCIPL Thickness** | **Asymmetry in Relative GCIPL Thickness** |
| --- | --- | --- | --- | --- | --- | --- |
| Superior hemifield | | | | | | |
| Clusters 6-8 (central) | 0.16 (<0.0001) | -0.003 (<0.0001) | 0.011 (<0.0001) | -0.007 (<0.0001) | -0.011 (<0.0001) | -0.007 (<0.0001) |
| Clusters 4-5 | 0.42 (<0.0001) | -0.007 (<0.0001) | -0.006 (0.18) | -0.014 (<0.0001) | -0.023 (<0.0001) | -0.012 (<0.0001) |
| Cluster 3 | 0.81 (<0.0001) | -0.014 (<0.0001) | -0.044 (<0.0001) | -0.017 (<0.0001) | -0.026 (<0.0001) | -0.007 (0.0002) |
| Cluster 2 | 0.19 (0.12) | -0.004 (0.012) | 0.004 (0.62) | -0.023 (<0.0001) | -0.008 (0.010) | -0.016 (<0.0001) |
| Cluster 1 (peripheral) | 0.24 (0.013) | -0.006 (0.0003) | 0.004 (0.67) | -0.026 (<0.0001) | -0.001 (0.79) | -0.02 (<0.0001) |
| Inferior hemifield | | | | | | |
| Clusters 6-8 (central) | 0.1 (<0.0001) | -0.002 (<0.0001) | 0.01 (<0.0001) | 0.001 (0.063) | -0.01 (<0.0001) | 0.001 (0.068) |
| Clusters 4-5 | 0.45 (<0.0001) | -0.009 (<0.0001) | -0.002 (0.68) | 0.004 (0.006) | -0.03 (<0.0001) | 0.004 (0.002) |
| Cluster 3 | 0.76 (<0.0001) | -0.012 (<0.0001) | -0.034 (<0.0001) | -0.007 (0.0002) | -0.013 (<0.0001) | -0.007 (0.0004) |
| Cluster 2 | 0.11 (0.37) | -0.002 (0.26) | 0.002 (0.83) | -0.026 (<0.0001) | -0.01 (0.001) | -0.023 (<0.0001) |
| Cluster 1 (peripheral) | 0.26 (0.019) | -0.002 (0.30) | -0.007 (0.32) | -0.003 (0.47) | 0.003 (0.37) | -0.003 (0.50) |

SD, standard deviation; GCIPL, ganglion cell-inner plexiform layer thickness

**Supplementary Table 2.** Eigenvectors for the significant parameters per principal components regression, with blank cells (-) indicating those where the parameter was non-significant. Eigenvalues (λ) from principal components analyses containing the significant parameter estimates only are also included for each principal component (PC). Per convention, superior and inferior hemifields refer to visual field format.

|  | **Mean** | **SD** | **Asymmetry** | **Relative GCIPL Thickness** | **Asymmetry in Relative GCIPL Thickness** | **λ** |
| --- | --- | --- | --- | --- | --- | --- |
| Superior hemifield | |  |  |  |  |  |
| Clusters 6-8 (central) | 0.36 | -0.38 | 0.54 | 0.38 | 0.54 | 2.83 |
| Clusters 4-5 | 0.40 | - | 0.59 | 0.44 | 0.55 | 2.34 |
| Cluster 3 |  |  |  |  |  |  |
| PC1 | 0.46 | 0.24 | 0.57 | 0.47 | 0.43 | 2.26 |
| PC2 | -0.43 | -0.63 | 0.30 | -0.13 | 0.55 | 1.48 |
| Cluster 2 | 0.35 | - | 0.67 | 0.20 | 0.62 | 1.77 |
| Cluster 1 (peripheral) | 0.40 | - | 0.67 | - | 0.63 | 1.76 |
| Inferior hemifield | |  |  |  |  |  |
| Clusters 6-8 (central) | 0.55 | -0.43 | 0.39 | 0.60 | - | 1.54 |
| Clusters 4-5 | -0.69 | - | 0.27 | -0.60 | 0.29 | 2.17 |
| Cluster 3 |  |  |  |  |  |  |
| PC1 | 0.35 | 0.19 | 0.60 | 0.33 | 0.60 | 2.27 |
| PC2 | -0.58 | -0.68 | 0.30 | -0.10 | 0.31 | 1.36 |
| Cluster 2 | 0.25 | - | 0.67 | 0.21 | 0.67 | 2.02 |
| Cluster 1 (peripheral) | -0.71 | -0.71 | - | - | - | 1.25 |

SD, standard deviation; GCIPL, ganglion cell-inner plexiform layer thickness; PC1, principal component 1; PC2, principal component 2

**Supplementary Table 3.** Parameter estimates from principal components regression with predicted outcomes for relatively central hemi-clusters included, for the hemi-clusters where principal components analysis methods demonstrated a significant improvement over the more conventional percentile method. P values per parameter estimate are included in brackets. Per convention, superior and inferior hemifields refer to visual field format.

|  | **Intercept** | **Mean** | **SD** | **Asymmetry** | **Relative GCIPL Thickness** | **Asymmetry in Relative GCIPL Thickness** | **Predicted Outcome** | | | |
| --- | --- | --- | --- | --- | --- | --- | --- | --- | --- | --- |
|  |  |  |  |  |  |  | **Clusters 6-8** | **Clusters 4-5** | **Cluster 3** | **Cluster 2** |
| Superior hemifield | | | | | | | | | | |
| Clusters 4-5 | 0.34 (<0.0001) | -0.006 (<0.0001) | -0.003 (0.50) | -0.011 (<0.0001) | -0.019 (<0.0001) | -0.009 (<0.0001) | 0.11 (<0.0001) | N/A | N/A | N/A |
| Cluster 3 | 0.57 (<0.0001) | -0.01 (<0.0001) | -0.031 (<0.0001) | -0.01 (<0.0001) | -0.02 (<0.0001) | -0.003 (0.14) | 0.11 (<0.0001) | 0.11 (<0.0001) | N/A | N/A |
| Cluster 2 | 0.06 (0.57) | -0.008 (<0.0001) | 0.001 (0.93) | -0.006 (0.14) | -0.027 (<0.0001) | 0.003 (0.36) | 0.12 (<0.0001) | 0.16 (<0.0001) | 0.11 (<0.0001) | N/A |
| Cluster 1 (peripheral) | -0.02 (0.83) | -0.003 (0.048) | 0.008 (0.30) | -0.017 (<0.0001) | -0.011 (<0.0001) | -0.011 (0.0003) | 0.08 (<0.0001) | 0.11 (<0.0001) | 0.07 (<0.0001) | 0.1 (<0.0001) |
| Inferior hemifield | | | | | | | | | | |
| Clusters 4-5 | 0.34 (0.0001) | -0.006 (<0.0001) | -0.001 (0.80) | 0.004 (0.002) | -0.016 (<0.0001) | 0.004 (0.0008) | 0.09 (<0.0001) | N/A | N/A | N/A |
| Cluster 3 | 0.33 (<0.0001) | -0.006 (<0.0001) | -0.011 (<0.0001) | -0.004 (0.035) | -0.011 (<0.0001) | -0.004 (0.03) | 0.07 (<0.0001) | 0.07 (<0.0001) | N/A | N/A |
| Cluster 2 | 0.17 (0.16) | -0.006 (0.002) | -0.004 (0.57) | -0.009 (0.041) | -0.014 (<0.0001) | -0.009 (0.011) | 0.08 (<0.0001) | 0.08 (<0.0001) | 0.08 (<0.0001) | N/A |
| Cluster 1 (peripheral) | 0.11 (0.30) | -0.003 (0.12) | -0.003 (0.61) | 0.002 (0.71) | -0.006 (0.09) | 0.001 (0.73) | 0.08 (<0.0001) | 0.07 (<0.0001) | 0.09 (<0.0001) | 0.09 (<0.0001) |

SD, standard deviation; GCIPL, ganglion cell-inner plexiform layer thickness; N/A, not applicable

**Supplementary Table 4.** Eigenvectors for the significant parameters per principal components regression with predicted outcomes for relatively central hemi-clusters included, with blank cells (-) indicating those where the parameter was non-significant. Eigenvalues (λ) from principal components analyses containing the significant parameter estimates only are also included. Per convention, superior and inferior hemifields refer to visual field format.

|  | **Mean** | **SD** | **Asymmetry** | **Relative GCIPL Thickness** | **Asymmetry in Relative GCIPL Thickness** | **Predicted Outcome** | | | | **λ** |
| --- | --- | --- | --- | --- | --- | --- | --- | --- | --- | --- |
|  |  |  |  |  |  | **Clusters 6-8** | **Clusters 4-5** | **Cluster 3** | **Cluster 2** |  |
| Superior hemifield | |  |  |  |  |  |  |  |  |  |
| Clusters 4-5 | 0.39 | - | 0.51 | 0.38 | 0.45 | -0.48 | N/A | N/A | N/A | 2.89 |
| Cluster 3 | 0.41 | 0.25 | 0.38 | 0.43 | - | -0.47 | -0.47 | N/A | N/A | 3.23 |
| Cluster 2 | 0.32 | - | 0.28 | 0.36 | - | -0.49 | -0.46 | -0.49 | N/A | 2.66 |
| Cluster 1 (peripheral) | 0.23 | - | 0.29 | 0.09 | 0.18 | -0.46 | -0.40 | -0.45 | -0.51 | 3.18 |
| Inferior hemifield | |  |  |  |  |  |  |  |  |  |
| Clusters 4-5 |  |  |  |  |  |  |  |  |  |  |
| PC1 | 0.35 | - | 0.52 | 0.36 | 0.51 | -0.46 | N/A | N/A | N/A | 2.54 |
| PC2 | -0.49 | - | 0.47 | -0.34 | 0.49 | 0.43 | N/A | N/A | N/A | 1.39 |
| Cluster 3 | 0.40 | 0.21 | 0.33 | 0.35 | 0.34 | -0.48 | -0.47 | N/A | N/A | 3.23 |
| Cluster 2 | 0.26 | - | 0.26 | 0.35 | 0.28 | -0.48 | -0.47 | -0.46 | N/A | 3.33 |
| Cluster 1 (peripheral) | - | - | -0.07 | -0.10 | - | 0.50 | 0.49 | 0.50 | 0.49 | 3.29 |

SD, standard deviation; GCIPL, ganglion cell-inner plexiform layer thickness; PC1, principal component 1; PC2, principal component 2; N/A, not applicable

**Supplementary Table 5.** Thresholds derived from Youden’s criterion of the maximum sum of sensitivity and specificity, applied to the receiver operating characteristic curves (ROCCs) derived for each of the tested ganglion cell-inner plexiform layer (GCIPL)-based models in the ROCC cohort.

|  | **Percentile** | **PCA** | **PCA plus central** |
| --- | --- | --- | --- |
| Superior hemifield | | | |
| Clusters 6-8 (central) | 0.423 | 0.080 | N/A |
| Clusters 4-5 | 0.230 | 0.373 | 0.164 |
| Cluster 3 | 0.095 | 0.252 | 0.163 |
| Cluster 2 | 0.096 | 0.251 | 0.215 |
| Cluster 1 (peripheral) | 0.097 | 0.277 | 0.138 |
| Inferior hemifield | | | |
| Clusters 6-8 (central) | 0.130 | 0.097 | N/A |
| Clusters 4-5 | 0.183 | 0.084 | 0.123 |
| Cluster 3 | 0.018 | 0.274 | 0.318 |
| Cluster 2 | 0.074 | 0.192 | 0.427 |
| Cluster 1 (peripheral) | 0.074 | 0.257 | 0.290 |

N/A, not applicable

**Supplementary Table 6.** Sensitivities with 95% confidence intervals calculated using the optimal ganglion cell-inner plexiform layer (GCIPL)-based method in the receiver operating characteristic curve (ROCC) and test cohorts, stratified by diagnostic category. Per convention, superior and inferior hemifields refer to visual field format.

|  | **ROCC Cohort**  **Glaucoma** | **Test Cohort** | **Test Cohort** |
| --- | --- | --- | --- |
|  |  | **Suspect** | **Glaucoma** |
| Superior hemifield | | | |
| Clusters 6-8 (central) | 0.82 (0.70-0.95) | 0.00 (0.00-0.00) | 0.70 (0.51-0.88) |
| Clusters 4-5 | 0.90 (0.81-0.98) | 0.29 (0.13-0.44) | 0.88 (0.79-0.98) |
| Cluster 3 | 0.93 (0.87-1.00) | 0.49 (0.33-0.64) | 0.84 (0.73-0.94) |
| Cluster 2 | 0.86 (0.76-0.96) | 0.28 (0.11-0.44) | 0.72 (0.59-0.85) |
| Cluster 1 (peripheral) | 0.91 (0.83-0.99) | 0.35 (0.15-0.54) | 0.76 (0.62-0.91) |
| Inferior hemifield | | | |
| Clusters 6-8 (central) | 0.91 (0.79-1.00) | 0.56 (0.29-0.83) | 0.57 (0.36-0.77) |
| Clusters 4-5 | 0.81 (0.68-0.94) | 0.46 (0.30-0.63) | 0.42 (0.27-0.57) |
| Cluster 3 | 0.93 (0.87-1.00) | 0.49 (0.33-0.64) | 0.84 (0.73-0.94) |
| Cluster 2 | 0.72 (0.60-0.85) | 0.30 (0.17-0.44) | 0.66 (0.51-0.81) |
| Cluster 1 (peripheral) | 0.73 (0.61-0.85) | 0.39 (0.24-0.55) | 0.54 (0.38-0.70) |

**Supplementary Table 7.** Specificities with 95% confidence intervals calculated using the optimal ganglion cell-inner plexiform layer (GCIPL)-based method in the receiver operating characteristic curve (ROCC) and test cohorts, stratified by diagnostic category. Per convention, superior and inferior hemifields refer to visual field format.

|  | **ROCC Cohort** | | **Test Cohort** | | | |
| --- | --- | --- | --- | --- | --- | --- |
|  | **Healthy** | **Glaucoma** | **Healthy** | **Suspect** | **PPG** | **Glaucoma** |
| Superior hemifield | | | | | | |
| Clusters 6-8 (central) | 0.98 (0.96-1.00) | 0.61 (0.48-0.74) | 0.98 (0.97-1.00) | 0.94 (0.91-0.97) | 0.78 (0.70-0.87) | 0.60 (0.49-0.72) |
| Clusters 4-5 | 0.95 (0.91-0.99) | 0.38 (0.23-0.53) | 0.97 (0.95-1.00) | 0.82 (0.77-0.87) | 0.60 (0.50-0.70) | 0.52 (0.38-0.66) |
| Cluster 3 | 0.92 (0.88-0.97) | 0.44 (0.27-0.61) | 0.93 (0.90-0.97) | 0.68 (0.74-0.62) | 0.47 (0.36-0.57) | 0.57 (0.42-0.72) |
| Cluster 2 | 0.92 (0.88-0.97) | 0.45 (0.30-0.60) | 0.95 (0.92-0.98) | 0.79 (0.74-0.84) | 0.68 (0.58-0.78) | 0.60 (0.46-0.74) |
| Cluster 1 (peripheral) | 0.91 (0.86-0.96) | 0.43 (0.29-0.58) | 0.94 (0.91-0.98) | 0.80 (0.75-0.85) | 0.68 (0.58-0.78) | 0.49 (0.36-0.62) |
| Inferior hemifield | | | | | | |
| Clusters 6-8 (central) | 0.93 (0.89-0.98) | 0.34 (0.23-0.45) | 0.87 (0.83-0.92) | 0.62 (0.56-0.68) | 0.43 (0.33-0.54) | 0.38 (0.27-0.50) |
| Clusters 4-5 | 0.92 (0.88-0.97) | 0.43 (0.30-0.57) | 0.94 (0.91-0.97) | 0.77 (0.72-0.83) | 0.57 (0.46-0.67) | 0.38 (0.24-0.51) |
| Cluster 3 | 0.92 (0.88-0.97) | 0.44 (0.27-0.61) | 0.93 (0.90-0.97) | 0.68 (0.62-0.74) | 0.47 (0.36-0.57) | 0.57 (0.42-0.72) |
| Cluster 2 | 0.93 (0.89-0.98) | 0.84 (0.73-0.95) | 0.96 (0.93-0.99) | 0.86 (0.81-0.90) | 0.72 (0.62-0.81) | 0.83 (0.73-0.93) |
| Cluster 1 (peripheral) | 0.91 (0.86-0.96) | 0.82 (0.69-0.94) | 0.95 (0.92-0.98) | 0.82 (0.77-0.87) | 0.70 (0.61-0.80) | 0.74 (0.62-0.86) |

PPG, pre-perimetric glaucoma

**Supplementary Table 8.** Sensitivities and specificities with 95% confidence intervals calculated using the optimal GCIPL-based method in glaucoma suspects with two reliable visual field results from the same day (n=190), and an additional criterion that visual field defects within hemi-clusters needed to be repeatable across both attempts.

|  | **Sensitivity** | **Specificity** |
| --- | --- | --- |
| Clusters 6-8 (central) | 0.00 (0.00-0.00) | 0.98 (0.96-1.00) |
| Clusters 4-5 | 0.11 (0.00-0.32) | 0.95 (0.92-0.98) |
| Cluster 3 | 0.11 (0.00-0.32) | 0.94 (0.91-0.98) |
| Cluster 2 | 0.27 (0.01-0.54) | 0.95 (0.91-0.98) |
| Cluster 1 (peripheral) | 0.00 (0.00-0.00) | 0.97 (0.95-1.00) |
| Clusters 6-8 (central) | 0.50 (0.00-1.00) | 0.99 (0.98-1.00) |
| Clusters 4-5 | 0.30 (0.02-0.58) | 0.95 (0.92-0.98) |
| Cluster 3 | 0.38 (0.17-0.59) | 0.91 (0.86-0.95) |
| Cluster 2 | 0.47 (0.25-0.70) | 0.94 (0.9-0.97) |
| Cluster 1 (peripheral) | 0.40 (0.15-0.65) | 0.94 (0.9-0.97) |
